# Contextual factors influencing the use of digital health by patients and physicians in primary care: a scoping review protocol

**DOI:** 10.1136/bmjopen-2024-088169

**Published:** 2024-12-20

**Authors:** Katarzyna Luchowska, Małgorzata Chmielewska, Katarzyna Byszek, Agata Olearczyk, Anna Gawrońska, Barbara Więckowska

**Affiliations:** 1 Health Innovation Unit, SGH Warsaw School of Economics, Warsaw, Poland

**Keywords:** eHealth, Physicians, Patients, Primary Health Care

## Abstract

**Abstract:**

**Introduction:**

Integrating digital health technologies to improve the overall performance of healthcare systems counts among the top priorities of the WHO. As evidenced in recent research, there are specific factors that may decisively determine the effective implementation and development of innovative digital health solutions. This review attempts to recognise and map the existing body of research and evidence to identify the determinants that either favour or hinder the deployment and use of digital health technologies by patients and physicians in primary care.

**Methods and analysis:**

The scoping review will be conducted in accordance with the JBI Manual for Evidence Synthesis (2024 edition introducing nine stages) and with the Preferred Reporting Items for Systematic Reviews and Meta-Analyses extension for Scoping Reviews checklist. Search strategy will be conducted in PubMed, Embase, Cochrane Database of Systematic Reviews and Scopus in November 2024. The review will include quantitative, qualitative, mixed-methods studies, systematic, scoping or umbrella reviews, as well as text and opinion papers. The study selection process will be conducted by two researchers using Rayyan. The data will be analysed using MAXQDA and extracted into a tool prepared by the researchers. This review will summarise the existing data and will help create a list of potential and determinant barriers and facilitators that may be useful in conducting empirical research on embedding new digital health solutions and sustaining their use.

**Ethics and dissemination:**

As no primary data will be collected for the purposes of this study, no formal ethical approval is required. Results will be published in a peer-reviewed journal. The findings will be used to conduct further research (HORIZON.2.1;grant:101095424) on the determinants of digital health implementation in primary care at the national level and to prepare recommendations for key stakeholders, as well as to develop recommendations on health policy in the European Union.

**STRENGTHS AND LIMITATIONS OF THIS STUDY**

- The review process will be conducted in accordance with JBI scoping review manual and PRISMA extension for a Scoping Review checklist (PRISMA-ScR).
- A detailed search strategy will cover a broad area of existing scientific evidence (quantitative, qualitative, mixed-methods studies, reviews, as well as opinion papers) as a support for further research.
- Search strategy will be conducted in four bibliographic databases, allowing the medical and the socio-economic perspective to be taken into account.
- The quality of the included studies or the risk of bias will not be assessed.
- Only papers in English or Polish published in the 5 years preceding the search date will be included.

## Introduction

 Among its core health priorities for 2020–2030, the WHO advocates to strengthen health systems through the use of digital health (DH) technologies to improve access to and the quality of care (including by means of innovative healthcare models) and the overall performance of the healthcare systems, thereby addressing the growing demand for healthcare worldwide.^1^

‘DH’ is a concept that invokes knowledge and the use of digital technologies to improve health. The WHO defines it in technical terms, as information and communication technologies (ICT) providing access to health services, education and research also referred to as eHealth,^1^ while the European Commission, in addition to the technical aspect, also recognises the importance of organisational improvements and cultural dimensions, such as the development of prohealth attitudes.^2^

In terms of how eHealth is applied in real healthcare settings, we can distinguish the following areas (to account for its administrative, clinical and educational aspects):

1. Telemedicine and telecare (remote consultation and remote patient monitoring).
2. Clinical information systems (electronic medical records, management and monitoring of clinical and institutional practices).
3. Information network (related to e-prescriptions and e-referrals).
4. Disease registries and non-clinical systems (used in public health/healthcare management).
5. Mobile health (m-health) (mobile technologies, including application—Apps).
6. Personalised health (p-health) (portable or implantable technologies used in therapy).
7. Big Data (large-scale data sources for a holistic view of individual patients and environmental factors affecting health data types and sources).^3^

With the progressing digital transformation, the role of DH is becoming increasingly strategic. It currently involves not only ICT solutions that support the delivery of healthcare services but also covers areas of fast-evolving advanced computer sciences, for example, advanced computing, big data, genomics or artificial intelligence.^1^

Primary care (PC) is the healthcare setting where DH needs to be embedded on a larger scale and due recognition should be given to the role of end users in this process^4 5^: a patient (understood as ‘a person who is a recipient of healthcare…’)^6^ and a physician—a healthcare provider.^7 8^ The concept of primary healthcare (PHC) evolved during the 1970s, when WHO and UNICEF developed the strategy for PHC as the means to achieve Health for All by the Year 2000.^9^ In 1978, at the International Conference on Primary Health Care in Alma-Ata participating countries signed a Declaration which established a new vision of PHC by defining it as the place providing evidence-based medical care, using socially acceptable methods and technologies, which, through its universal accessibility and appropriate cost, brings healthcare closer to the people, being their first point of contact with the national healthcare system.^7^ In 2015, a European Resolution was approved highlighting the need to transform health services in Europe to address the challenges of the 21st century, thus calling on Member States to adopt a proactive, patient-centred approach including better coordination in health promotion, disease prevention, healthcare and lifelong health management. The aim of the adopted Resolution was to improve quality of care, health outcomes and reduce health inequalities while addressing fundamental values such as solidarity, equality and participation.^7^

PC has traditionally been identified with general medicine, due to the presence of PC physicians.^10^ In some countries, doctors from other specialisations are considered as PHC teams’ members, for example: dentists (Belgium, Croatia, Germany, the UK and the USA) paediatricians (Czechia, France, Slovenia and the USA), gynaecologists (France, Czechia, Slovenia, the USA), ophthalmologists (France).^11^,^18^ OECD points out that PHC also relies on other healthcare professionals^19^ such as nurses (eg, Canada, Mexico, Norway, Poland and the USA), physiotherapists (eg, France, Germany, Norway, Slovenia and Sweden) or pharmacists (eg, Croatia, France, Germany, the UK and the USA)^20^,^24^ hence the term extended primary healthcare.^10^

In order to successfully implement innovative DH solutions in PC, it is essential that due consideration be given to contextual factors thought of as ‘strategic’ for achieving quality, efficiency and optimal use of existing resources.^25^ More broadly, contextual factors refer to the determinants that emerge during the implementation process, covering micro (important for individuals/teams; eg, individual perception), meso (organisational factors, eg, organisational culture) and macro (external environment, ie, politics) levels of the healthcare system. These factors are inter-related and coalesce into a multidimensional construct. Most studies reveal that the context captures determinants that are independent of the intervention, that is, characteristics of the intervention are distinct from characteristics of the context.^26^ However, Scott and Briggs^4^ are among those who advised not to underestimate the phenomenon of interconnection and interdependence of ‘social’ (eg, people, culture) and ‘technical’ (eg, equipment, technology and procedures) aspects when implementing digital innovations, specifically in the healthcare sector (considered highly ‘unpredictable and non-routine’ and involving many uncertainties)—a change in one element will affect the rest of the elements. Also, Paré *et al* revealed that project failure is only rarely attributed solely to technological factors.^27^ The dynamic nature of implementation, which is linked to the rapid evolution of the healthcare system in time, is embedded in the contemporary understanding of the context.^28^ In a narrower sense, contextual factors can be defined as facilitators and barriers that are revealed when innovations are implemented or used.^29 30^ According to the WHO recommendations on DH implementation strategies, the national context should be considered to take into account the local health priorities, the level of DH advancement, the relevant needs and the existing resource constraints.^1^

With regard to factors influencing the use of DH, after an initial review of the literature (including systematic as well as scoping reviews), no systematic study was found by authors of this paper that identified a list of contextual factors influencing the application/use of DH in PC by both clinicians and patients.

The available reviews often focus on the general use of DH in healthcare, without addressing the specificity of its use in PHC.^30^,^37^ Attention is given to the application of digital solutions (a) in specific disease entities,^31 34 35^ (b) in terms of patient age and gender,^32 36^ (c) from the perspective of the experience/attitude by the population of healthcare professionals towards innovations^30^ or taking into account and (d) country specificities.^36^

When factors influencing the use of DH in PC are analysed, innovations are narrowed down to single technologies or interventions, for example, telemedicine^38^,^49^ or remote monitoring systems^50^ being DH domains. In addition, inquiries are being made into the unravelling of determinants during the usage/utilisation of digital innovations in specific populations, such as adult patients,^39^ people over 65 and their carers and staff involved in the care of this patient group,^42^ people from specific population groups,^44^ patients with diabetes,^46 49^ burdened with multimorbidity or at least one chronic disease,^50^ healthcare professionals dealing with a specific disease entity^41 45 49^ or refer to the population of a selected area, for example, countries with a specific income level.^38 51^

The number of systematic reviews found in the initial search indicates the importance of the context within the use of digital innovations in healthcare, that is, cultural factors, environmental factors and geographical location.^30 32 33^ Some of them point to the existence of hundreds of factors that constitute barriers or facilitators affecting the DH uptake, hence grouping them, categorise them or create models.^30 32 33^ Schreiweis *et al*, Borges do Nascimento *et al* and Moulaei *et al* noted that not all of these factors are equally important.^30 32 33^ Schreiweis *et al* emphasised the importance depending on the specific stakeholders or area of application,^33^ Ward *et al* and Jonnagaddala *et al* suggested that factors observed during epidemic periods may not be determinant in conditions of reduced risk of disease spread.^43 48^ Dhyani *et al*, on the other hand, points out that it is the combination of multilevel factors that influences implementation and utilisation in different contexts, emphasising that while some factors may be common, for example, for different geographical area, they are characterised by their contextual in nature.^51^ Also Moulaei *et al* noted that some factors may be general/universal in nature, while some may be dependent on specific characteristics, for example, of a particular population,^32^ whereas Wittenberg *et al* made telemedicine implementations dependent on culturally sensitive aspects.^52^ Other reviews, which have studied physician and patient populations simultaneously, indicate that quite different factors may be acting on both sides.^40 47 48^ In addition, Dhyani *et al* pointed out the need to study not only patient or physician factors, but also the interactions between them.^51^ Conway *et al* highlighted that timely identification of these factors can contribute to faster implementation of DH interventions and increase the likelihood of adoption.^53^ Comprehensive frameworks can be helpful in elucidating factors influencing utilisation and adoption.^26 54^ The reviews also remark that findings often relate only to the implementation or initial use of DH, making information on factors influencing DH utilisation at different stages a research gap.^34 55^ Greenhalgh and Abimbola^28 56^ argued that the adoption of promising interventions has yet to be examined using a systematic approach, and propose using the NASSS framework to assess the reasons for ‘non-adoption, abandonment of technologies’, and the challenges related to the advancement stage of such technologies in healthcare organisations, such as ‘scale-up, spread and sustainability’.

This review is complementary to and supplements the existing body of knowledge about the determinants of the use of DH in PC worldwide. It primarily seeks to identify and map existing research and evidence on the key factors related to the adoption of DH by patients and physicians in PC.

This review can answer the following research questions:

1. What are the factors that have the potential to facilitate or hinder the use of digital health?
2. Which facilitators and barriers have a determining impact on the use of digital health?
3. What is the current state of research and evidence (both qualitative and quantitative) that explores the impact of facilitators and barriers on the use of digital health?

## Methods and analysis

The scoping review will be conducted in accordance with the Joanna Briggs Institute (JBI) methodology for scoping reviews^57^ and with the Preferred Reporting Items for Systematic Reviews and Meta-Analyses extension for Scoping Reviews (PRISMA-ScR) checklist.^58^ Our review will follow scoping review framework consisting of nine consecutive stages: (1) defining and aligning the objective and questions, (2) developing and aligning the inclusion criteria with the objective and questions, (3) describing the planned approach to evidence searching, selection, data extraction and presentation of the evidence, (4) searching for the evidence, (5) selecting the evidence, (6) extracting the evidence, (7) analysis of the evidence, (8) presentation of the results and (9) summarising the evidence in relation to the purpose of the review, making conclusions and noting any implications of the findings.^57 59 60^ The protocol will be registered with Open Science Framework (https://osf.io/) and the review will be performed in 2024.

### Stage 1: defining and aligning the objective and questions

We posed the following specific research questions (RQ):

RQ1: What types of studies are available?

RQ2: What is the main focus of the available studies?

RQ3: What are the theoretical frameworks/models based on which factor studies are conducted?

RQ4: What factors (facilitators and barriers) have been described as influencing DH usage?

RQ5: What is the noted variation in factors across: countries/domains of DH/epidemic/process of DH use/physician and patient population?

### Stage 2: developing and aligning the inclusion criteria with the objective and questions

The inclusion criteria were developed based on the PCC framework (population, concept and context) (table 1).

**Table 1:** PCC framework for eligibility studies

PCC element	Inclusion criteria
Population	Patients and physicians (general practitioners)
Concept	Factors influencing the use of digital health
Context	Primary healthcare

This scoping review will consider both qualitative and quantitative studies. In addition, systematic, scoping or umbrella reviews that meet the inclusion criteria will also be considered. Text and opinion papers will also be under consideration for inclusion in this scoping review.

Due to the significant advances in DH and the dynamic development caused by the COVID-19 pandemic, only papers published in the 5 years preceding the search date will be included in this review.

Book chapters, conference abstracts, protocols, comment letters, preprints or publications in other language than English or Polish will be excluded from this scoping review.

### Stage 3: describing the planned approach to evidence searching, selection, data extraction and presentation of the evidence

As indicated in this step, it should include planning for the search strategy, selection, extraction and presentation of the outcomes, which should be reflected in the protocol.^59^ Details of the various elements are discussed in the following subsections.

### Stage 4: searching for the evidence

This review is based on a three-step search strategy. The first step involved an initial search of studies in MEDLINE via PubMed to identify articles on the topic and relevant keywords. In order to build a full search strategy, we use keywords from the publications found, as well as words entering the search strategies in the scope and systematic reviews found. The search strategy merges terms from four topics: (1) population (patients, physicians)/patients or physicians (2) factors (3) DH (4) PC (table 2) and will be adapted for each included database. The second step is to perform a comprehensive literature search in four databases. It includes the following databases: PubMed, Embase, Cochrane Database of Systematic Reviews and Scopus. As a final step, reference lists of publications meeting the inclusion criteria will be screened to identify other relevant studies. Finally, an update search will be performed.

**Table 2:** Search terms for the databases

PCC element	Topic	Search terms
Population	Patients	Patient*OR Citizen*
	Physicians	Physician*OR Doctor*
Concept	Factors	Factor* OR Barrier* OR Facilitator* OR Enabler* OR Benefit* OR Opportunity OR Opportunities OR Challenge* OR Affect OR Impact OR Reason OR Circumstance OR Cause OR Context OR Influence OR Conditions OR Surroundings OR Determinant OR Stimulus OR Incentive OR Motivation OR Encouragement OR Difficulty OR Restriction OR Limitation OR Support OR Driver
	Digital health (with domains):	Digital health OR Digital health care OR
	eHealth:	eHealth OR e-Health OR Electronic health OR information and communication technologies (ICTs) OR Telematic OR
	Telemedicine/telecare:	Telemedicine/Tele-medicine/Tele medicine OR Telecare/Tele care OR Telehealth/Tele health OR Teleconsulting OR Teleconsultation* OR Digital consultation* OR Remote consultation* OR Remote medical consultation OR Electronic consultation*/e-consultation*/e-consult* OR Electronic medical consultation* OR Satellite consultation* OR Satellite medical consultation* OR Online/video/phone/chat consultation* OR Online visit* OR eVisit/e-
		Visit OR Remote health care service* OR Remote diagnosis OR Tele-diagnosis OR Remote management OR Telemanagement OR Remote clinic OR Satelite clinic OR Virtual models of care OR Virtual care OR Virtual provider OR Virtual consulting OR Telehealth platform* OR
	Clinical information systems:	Electronic health (care) record*/data OR Electronic medical record*/data OR Electronic patient record*/data OR EHR OR Personal health (care) record/data OR Personal medical record OR PHR OR Patient access to records OR Medical records OR Medical informatics OR
	Information networks:	e-referrals OR e-prescribing OR Patient portal* OR patient web portal* OR Information system* OR Digital healthcare platform* OR
	Disease registries and other non-clinical systems:	Health information system* OR Biomedical informatics OR Health informatics OR
	Mobile health:	Mobile health OR m-health OR mHealth OR mHealth technologies OR
	Personalised health:	Personalized health OR p-health OR phealth OR
	Advanced computing science:	Big data OR Genomics OR Artificial intelligence
Context	Primary healthcare	Primary health care OR Primary healthcare OR Access to primary health care OR Primary care OR Primary medical care OR Family medicine OR Family practic* OR Family caregiver* OR Family physician OR General practitioner* OR General Practice* OR General Practic* OR General medical practice OR GP*

### Stage 5: selecting the evidence

All identified citations during search will be collated and imported into Rayyan to remove duplicates and to conduct study selection process.

In a first step, a pilot selection process will be carried out in order to achieve high compatibility—each reviewer will be asked to classify 10 abstracts. In the second step, titles and abstracts will be screened by two independent reviewers. In case of disagreement, disagreements will be resolved through discussion and, in the absence of consensus, with the involvement of a third reviewer. In the third step, the full text of selected citations will also be evaluated by two independent reviewers. Reasons for exclusion of full-text papers do not meet the eligibility criteria will be noted and reported. Any inconsistencies between the two reviewers will be solved through discussion. If there is no consensus, disagreements will be resolved by a third reviewer. The final scoping review will include a PRISMA-ScR flow diagram reflecting the publication selection process.

### Stage 6: extracting the evidence

Data from included studies will be analysed with MAXQDA V.24.2.0 and extracted into a tool prepared by reviewers in the form of Microsoft Office Excel spreadsheet. They will be extracted by two independent reviewers. The data extracted will contain detailed information that is relevant to the research questions. An outline of the data collection is given in table 3.

**Table 3:** The outline of the data collection

Review question	Data to be extracted	Coding examples
RQ1	Authors/title	N/A
	Year of the publication	N/A (5 years and update)
	Research design	Empirical study (quantitative, qualitative, mix, others)
RQ2	Main objective	Measuring directly or indirectly the factors influencing the use of DH
RQ3	Theoretical framework/model	For example, Unified Theory of Acceptance and Use of Technology, Model of Behaviour Change, List of barriers and success factors created by Schreiweis *et al.*^33^
RQ4	Characteristic of factors (facilitators and barriers) affecting the DH usage	Potential—for example, exerts’ opinion, list of factors Determinising—for example, statistical significance
RQ5	Country	N/A (list of countries)
	Domain of DH	Telemedicine and telecare; clinical information systems; Information network (eg, e-prescriptions, e-referrals); disease registries and non-clinical systems generally used in public health/healthcare management; mobile health; personalised health; advanced computer sciences (eg, big data, artificial intelligence)
	Study circumstances	Epidemic/non-epidemic Disease
	Process of using	Implementation/utilisation
	Characteristic of population	Physicians: Specialisation Vocation experience Digital experience/use experience Patients and their caregivers: Health problem Age Digital experience/use experience

DHdigital healthN/Anot available

The team will pilot extraction form on the first 10 articles. If necessary, the draft data extraction tool will be adjusted and revised in an iterative manner during the whole process of extracting data.

One of the reviewers will write out the data and the other will check its suitability. Any incompatibility between the reviewers will be resolved through discussion, or in case of lack of consensus with an additional reviewer. If any information is missing or needs to be supplemented, the authors of the publication will be contacted for further clarification.

Studies included in the review will not be assessed critically or for risk of bias as the as the purpose of this review focuses on synthesising and outlining the range of evidence.

### Stage 7: analysis of the evidence

The following approaches to data analysis will be used in this review: descriptive statistics, narrative analysis and basic qualitative content analysis as this review intends to group factors influencing use of DH. Standard categories will be used for the types of empirical studies (quantitative, qualitative and mix). DH domains will be assigned in accordance with eHealth categorisation with the expansion made by WHO to include advanced computing sciences.^1 3^ Where the study in focus relates to a pandemic period, the disease type will be taken from the paper and the paper itself classified as ‘epidemic’. Other papers will be classified as ‘non-epidemic’. The population will be dichotomously divided into patients and physicians. Other data will not be classified and will be extracted from the publication (eg, country and stage of utilisation process). If necessary, the research team will consider and verify the coding template. An outline of the planned analysis approach regarding the specific research questions is presented in table 4.

**Table 4:** The outline of the planned analysis approach

Review question	Planned analysis approach
RQ1	Descriptive statistics
RQ2	Narrative analysis
RQ3	Narrative analysis
RQ4	Descriptive statistics and narrative analysis
RQ5	Descriptive statistics and basic qualitative content analysis

### Stage 8: presentation of the results

The analysed data will be mapped and presented visually. An outline of the planned presentation of the results regarding the specific research questions is presented in table 5.

**Table 5:** The outline of the planned presentation of the results

Review question	Planned presentation of the results
RQ1	Tabular format and/or waffle chart of the methodology used within the included evidence sources
RQ2	Tabular format
RQ3	Tabular format/mind map
RQ4	Tabular format
RQ5	Pie charts on maps showing variation in factors across countries Cross-tabulation/heat map/sunburst diagram showing factors specific to different domains of digital health included within the evidence sources Cross-tabulation/heat map/sunburst diagram showing factors specific to epidemics Cross-tabulation/heat map/ sunburst diagram showing factors specific to process of digital health use Cross-tabulation/heat map/sunburst diagram showing factors specific to different populations

### Stage 9: summarising the evidence in relation to the purpose of the review, making conclusions and noting any implications of the findings

This review will summarise data on factors (potential and determining) influencing use of DH in PHC both by patients and physicians. It will help to create a list of barriers and facilitators, both potential and determinant, that may be useful in conducting empirical research on a country-specific basis, introducing new DH solutions, as well as in sustaining utilisation. The review will also provide an outline of methodological issues (publication year, study design, main objective and theoretical framework/model) as well as mapping information on the countries in which the studies were conducted, the DH domains examined, the study environment, the circumstances under which the studies were conducted, the stage in the process of use and the characteristics of the populations studied. The review will answer the question of whether the above issues differentiate factors influencing the use of DH in PC.

### Patient and public involvement

At no stage in the creation of the scoping review was public or patient involvement considered.

## Ethics and dissemination

As no primary data will be collected for the purposes of this study, no formal ethical approval is required. Results will be published in a peer-reviewed journal. The findings will also be used to conduct further empirical studies on the determinants of DH implementation in PC at the national level and to prepare recommendations for key stakeholders (ministry of health and healthcare providers). They will also be disseminated through conference presentations. In a situation where the protocol requires changes after publication, any detailed clarifications will be reported.

## References

[1] World Health Organization (2021). Global strategy on digital health 2020-2025.

[2] European Commission Public health. https://health.ec.europa.eu/index_en.

[3] Cowie MR, Bax J, Bruining N (2016). e-Health: a position statement of the European Society of Cardiology. Eur Heart J.

[4] Scott PJ, Briggs JS (2010). STAT-HI: A socio-technical assessment tool for health informatics implementations. Open Med Inform J.

[5] Vasanthan L, Natarajan SK, Babu A (2024). Digital health interventions for improving access to primary care in India: A scoping review. PLOS Glob Public Health.

[6] World Health Organization (2009). Conceptual framework for the international classification for patient safety. https://iris.who.int/handle/10665/70882,%20https://www.who.int/publications/i/item/WHO-IER-PSP-2010.2.

[7] World Health Organization (2018). From Alma-Ata to Astana: Primary health care - reflecting on the past, transforming for the future Interim Report from the WHO European Region. https://www.who.int/docs/default-source/primary-health-care-conference/phc-regional-report-europe.pdf.

[8] World Health Organization (2018). Classification of Digital Health Interventions, Geneva.

[9] Quah SR (2017). International Encyclopedia of Public Health | ScienceDirect. https://www.sciencedirect.com/referencework/9780128037089/international-encyclopedia-of-public-health.

[10] Bourgueil Y, Marek A, Mousquès J (2009). Trois modèles types d’organisation des soins primaires en Europe, au Canada, en Australie et en Nouvelle-Zélande. Q éco santé.

[11] Gerkens S, Merkur S (2020). Belgium: Health System Review. Health Syst Transit.

[12] Dzakula A, Vočanec D, Banadinovic M (2021). Croatia: Health System Review. Health Syst Transit.

[13] Blümel M, Spranger A, Achstetter K (2020). Germany: Health System Review. Health Syst Transit.

[14] Anderson M, Pitchforth E, Edwards N (2022). United Kingdom: Health System Review. Health Syst Transit.

[15] Rice T, Rosenau P, Unruh LY (2020). United States: Health System Review. Health Syst Transit.

[16] Bryndová L, Šlegerová L, Votápková J (2023). Czechia: Health System Review. Health Syst Transit.

[17] Or Z, Gandré C, Seppänen A-V (2023). France: Health System Review. Health Syst Transit.

[18] Albreht T, Polin K, Pribaković Brinovec R (2021). Slovenia: Health System Review. Health Syst Transit.

[19] OECD Primary Care.

[20] Marchildon GP, Allin S, Merkur S (2020). Canada: Health System Review. Health Syst Transit.

[21] González Block MÁ, Reyes Morales H, Hurtado LC (2020). Mexico: Health System Review. Health Syst Transit.

[22] Saunes IS, Karanikolos M, Sagan A (2020). Norway: Health System Review. Health Syst Transit.

[23] Obwieszczenie Ministra Zdrowia z dnia 16 czerwca 2023 r (2023). w sprawie ogloszenia jednolitego tekstu rozporządzenia Ministra Zdrowia w sprawie świadczeń gwarantowanych z zakresu podstawowej opieki zdrowotnej.

[24] Janlöv N, Blume S, Glenngård AH (2023). Sweden: Health System Review. Health Syst Transit.

[25] Ploeg J, Wong ST, Hassani K (2019). Contextual factors influencing the implementation of innovations in community-based primary health care: the experience of 12 Canadian research teams. *Prim Health Care Res Dev*.

[26] Rogers L, De Brún A, McAuliffe E (2020). Defining and assessing context in healthcare implementation studies: a systematic review. BMC Health Serv Res.

[27] Paré G, Sicotte C, Jaana M (2008). Prioritizing the risk factors influencing the success of clinical information system projects. A Delphi study in Canada. Methods Inf Med.

[28] Greenhalgh T, Abimbola S (2019). The NASSS Framework - A Synthesis of Multiple Theories of Technology Implementation. Stud Health Technol Inform.

[29] Coles E, Anderson J, Maxwell M (2020). The influence of contextual factors on healthcare quality improvement initiatives: a realist review. Syst Rev.

[30] Borges do Nascimento IJ, Abdulazeem H, Vasanthan LT (2023). Barriers and facilitators to utilizing digital health technologies by healthcare professionals. NPJ Digit Med.

[31] Brands MR, Gouw SC, Beestrum M (2022). Patient-Centered Digital Health Records and Their Effects on Health Outcomes: Systematic Review. *J Med Internet Res*.

[32] Moulaei K, Moulaei R, Bahaadinbeigy K (2023). Barriers and facilitators of using health information technologies by women: a scoping review. BMC Med Inform Decis Mak.

[33] Schreiweis B, Pobiruchin M, Strotbaum V (2019). Barriers and Facilitators to the Implementation of eHealth Services: Systematic Literature Analysis. J Med Internet Res.

[34] Svendsen MJ, Wood KW, Kyle J (2020). Barriers and facilitators to patient uptake and utilisation of digital interventions for the self-management of low back pain: a systematic review of qualitative studies. BMJ Open.

[35] Tarver WL, Haggstrom DA (2019). The Use of Cancer-Specific Patient-Centered Technologies Among Underserved Populations in the United States: Systematic Review. *J Med Internet Res*.

[36] Walle AD, Demsash AW, Adem JB (2023). Exploring facilitators and barriers of the sustainable acceptance of e-health system solutions in Ethiopia: A systematic review. PLoS One.

[37] Wilson J, Heinsch M, Betts D (2021). Barriers and facilitators to the use of e-health by older adults: a scoping review. BMC Public Health.

[38] Abrokwa SK, Ruby LC, Heuvelings CC (2022). Task shifting for point of care ultrasound in primary healthcare in low- and middle-income countries-a systematic review. EClinMed.

[39] Campbell K, Greenfield G, Li E (2023). The Impact of Virtual Consultations on the Quality of Primary Care: Systematic Review. J Med Internet Res.

[40] Darley S, Coulson T, Peek N (2022). Understanding How the Design and Implementation of Online Consultations Affect Primary Care Quality: Systematic Review of Evidence With Recommendations for Designers, Providers, and Researchers. J Med Internet Res.

[41] Gonçalves RL, Pagano AS, Reis ZSN (2023). Usability of Telehealth Systems for Noncommunicable Diseases in Primary Care From the COVID-19 Pandemic Onward: Systematic Review. *J Med Internet Res*.

[42] Ilali M, Le Berre M, Vedel I (2023). Telemedicine in the primary care of older adults: a systematic mixed studies review. *BMC Prim Care*.

[43] Jonnagaddala J, Godinho MA, Liaw S-T (2021). From telehealth to virtual primary care in Australia? A Rapid scoping review. Int J Med Inform.

[44] Parker RF, Figures EL, Paddison CA (2021). Inequalities in general practice remote consultations: a systematic review. BJGP Open.

[45] Patil R, Shrivastava R, Juvekar S (2021). Specialist to non-specialist teleconsultations in chronic respiratory disease management: A systematic review. J Glob Health.

[46] Robson N, Hosseinzadeh H (2021). Impact of Telehealth Care among Adults Living with Type 2 Diabetes in Primary Care: A Systematic Review and Meta-Analysis of Randomised Controlled Trials. Int J Environ Res Public Health.

[47] Thiyagarajan A, Grant C, Griffiths F (2020). Exploring patients’ and clinicians’ experiences of video consultations in primary care: a systematic scoping review. BJGP Open.

[48] Ward K, Vagholkar S, Sakur F (2022). Visit Types in Primary Care With Telehealth Use During the COVID-19 Pandemic: Systematic Review. JMIR Med Inform.

[49] Zurynski Y, Carrigan A, Meulenbroeks I (2023). Transition models of care for type 1 diabetes: a systematic review. BMC Health Serv Res.

[50] Peyroteo M, Ferreira IA, Elvas LB (2021). Remote Monitoring Systems for Patients With Chronic Diseases in Primary Health Care: Systematic Review. JMIR Mhealth Uhealth.

[51] Dhyani VS, Krishnan JB, Mathias EG (2023). Barriers and facilitators for the adoption of telemedicine services in low-income and middle-income countries: a rapid overview of reviews. BMJ Innov.

[52] Wittenberg E, Goldsmith JV, Chen C (2021). Opportunities to improve COVID-19 provider communication resources: A systematic review. Pat Educ Couns.

[53] Conway A, Ryan A, Harkin D (2023). A review of the factors influencing adoption of digital health applications for people living with dementia. Dig Health.

[54] James HM, Papoutsi C, Wherton J (2021). Spread, Scale-up, and Sustainability of Video Consulting in Health Care: Systematic Review and Synthesis Guided by the NASSS Framework. J Med Internet Res.

[55] Shin HD, Durocher K, Sequeira L (2023). Information and communication technology-based interventions for suicide prevention implemented in clinical settings: a scoping review. BMC Health Serv Res.

[56] Scott P, Keizer N, Georgiou A (2019). Applied interdisciplinarytheory in health informatics: a knowledge base for Practitioners. IOS Press.

[57] Peters MDJ, Godfrey C, McInerney P (2020). JBI Manual for Evidence Synthesis. JBI. J B I.

[58] Tricco AC, Lillie E, Zarin W (2018). PRISMA Extension for Scoping Reviews (PRISMA-ScR): Checklist and Explanation. Ann Intern Med.

[59] Pollock D, Davies EL, Peters MDJ (2021). Undertaking a scoping review: A practical guide for nursing and midwifery students, clinicians, researchers, and academics. J Adv Nurs.

[60] Pollock D, Peters MDJ, Khalil H (2022). Recommendations for the extraction, analysis, and presentation of results in scoping reviews. JBI Evd Synth.

