# Supplementary material for: Contextual factors influencing the use of digital health by patients and physicians in primary care: a scoping review protocol
Source: BMJ Open. 2024 Dec 20;14(12):e088169. doi: 10.1136/bmjopen-2024-088169 (PMC11667403; doi:10.1136/bmjopen-2024-088169)
Supplement: Supplementary file 1 [file bmjopen-2024-088169.reviewer_comments.pdf]

PEER REVIEW HISTORY

BMJ Open publishes all reviews undertaken for accepted manuscripts. Reviewers are asked to complete a checklist review form and are provided with free text boxes to elaborate on their assessment. These free text comments are reproduced below.

ARTICLE DETAILS

Title (Provisional)

Contextual factors influencing the use of digital health by patients and physicians in primary care - a scoping review protocol

Authors

Luchowska, Katarzyna; Chmielewska, Małgorzata; Byszek, Katarzyna; Olearczyk, Agata; Gawrońska, Anna; Więckowska, Barbara

---

VERSION 1 - REVIEW

**Reviewer**	**1**
**Name**	**Kamalakannan, Sureshkumar**
**Affiliation**	**Northumbria University, Department of Social Work**
**Date**	**23-Jul-2024**
**COI**	**None**

This is a critical topic to be reviewed and I congratulate the authors of this manuscript. There is a need to transparently report key methodological details. I have provided my annotated comments on the manuscript and request the authors to revise it accordingly. Best Wishes.

****** The reviewer provided a marked copy with additional comments. Please contact the publisher for full details.**

**Reviewer**	**2**
**Name**	**Scott, Philip**
**Affiliation**	**University of Wales Trinity St David, Institute of Management and Health**
**Date**	**12-Aug-2024**
**COI**	**None**

This is a well designed study with a clearly explained and justified protocol. My only reservations are (1) that the very broad and high level nature of the "factors" may lead to very vague, woolly and diverse conclusions which may end up not adding very much, (2)

there is no mention of the sociotechnical perspective, which has a rich literature in this topic area (e.g. https://www.ncbi.nlm.nih.gov/pmc/articles/PMC3096986/), and (3) there is no acknowledgement of some very relevant earlier work (albeit described as risk factors, rather than just 'factors affecting use'):

https://ebooks.iospress.nl/publication/51886 https://pubmed.ncbi.nlm.nih.gov/18473092/

VERSION 1 - AUTHOR RESPONSE

Reviewer: 1
Dr. Sureshkumar Kamalakannan, Northumbria University
Comments to the Author:
This is a critical topic to be reviewed and I congratulate the authors of this manuscript. There is a need to transparently report key methodological details. I have provided my annotated comments on the manuscript and request the authors to revise it accordingly. Best Wishes.

1. Rather than patients - Other appropriate terms can be used. Respecting the service users of primary care. For example - Pregnant women who access these services are not patients because they are pregnant.
*The WHO definition of the term "patient" was added on page 5, line 135: "**A patient is a person who is a recipient of healthcare**, itself defined as services received by individuals or communities to promote, maintain, monitor or restore health. Patients are referred to rather than clients, tenants or consumers, although it is recognized that may recipients such as a health pregnant woman or a child undergoing immunization may not be regarded, or regard themselves, as patients. Healthcare includes self-care. Health, as defined by the World Health Organization, is the "state of complete physical, mental and social well-being and not merely the absence of disease or infirmity".*

- *World Health Organization & WHO Patient Safety. (2010). Conceptual framework for the international classification for patient safety version 1.1: final technical report January 2009. World Health Organization. https://iris.who.int/handle/10665/70882*
- *https://www.who.int/publications/i/item/WHO-IER-PSP-2010.2*

The understanding of the term "patient" is similar in other sources, including:

- *https://www.healthcareexcellence.ca/en/resources/engaging-patients-in-patient-safety-a-canadian-guide/glossary-of-terms/*
- *How to plan and conduct telehealth consultations with children and adolescents and their families. Geneva: World Health Organization; 2021. Licence: CC BY-NC-SA 3.0 IGO.*
- *Costa, Daniel S J et al. "Patient, client, consumer, survivor or other alternatives? A scoping review of preferred terms for labelling individuals who access healthcare*

across settings." BMJ open vol. 9,3 e025166. 7 Mar. 2019, doi:10.1136/bmjopen-2018-025166

2. Good to make these steps specific to the review. Currently it is very generic. It also misses important components about who when and how.
The abstract was rewritten according to the suggestions (page 2, lines: 44, 46-48, 56-61)

3. Before this section a short paragraph on the expected outcomes of this review could be added.
These amendments were introduced as suggested (lines: 58-61 on the page 2).

4. Please include a sentence or two explaining what this means to the service users, - it is prevention, promotion, rehabilitation etc. Looking only from service providers perspectives may narrow down the objectives of the review.
Selected amendments were introduced (lines: 144-150 on the page 5) – thank you very much for the suggestions.

5. This sentence need to be cited.
This is the authors' conclusion; appropriate additions were made accordingly in line 189, page 6.

6. Please cite this sentence.
The citation was supplemented (line 211 on page 7).

7.This is complex. There has been a very clear explanation of what constitutes the context and narrowing it down to just PH would not be helpful. Especially with the current definition which does not include what is means to be a consumer of PH. Please describe.
Taking into account the following two aspects of the guidelines:

1. *PCC (Population (or participants)/Concept/Context) framework is recommended by JBI to identify the main concepts in the primary review questions.*

- *Pollock, Danielle et al, 'Recommendations for the Extraction, Analysis, and Presentation of Results in Scoping Reviews' (2023) 21(3) JBI evidence synthesis 520*

2. ***The "Context"** element of a scoping review will vary depending on the objective/s and question/s of the review. The context should be clearly defined and may include, but is not limited to, consideration of cultural factors, such as geographic location and/or specific social, cultural, or gender-based interests. In some cases, context may also encompass*

*details about the specific setting (such as acute care, **primary health care** or the community). Reviewers may choose to **limit the context** of their review to a particular country or health system or healthcare setting, depending on the topic and objectives.*

- *https://beckerguides.wustl.edu/scopingreviews/PCC*

*primary care was indicated as the research area at this stage of the protocol, which is also mentioned in the title: "*Contextual factors influencing the use of digital health by patients and physicians **in primary care"***.*
The definition of PH was added as suggested (lines: 144-150 on the page 5) .

8. This again is tricky and require the purpose of including these designs and how the information from these designs will be synthesised together to answer the questions.
The information will be synthesized according to stage 6, as detailed in Table 3. The method of analysis was described in Stage 7 - Analysis of the evidence.

9. The use of various terms for this concept need to be described clearly to help understand how broad the scoping exercise is. Just using this term as a concept may not provide information to scope on this objective.
The understanding of the term "patient" was made clear as above.

10. Again this is very narrow. Need to include databases that include these kinds of studies. Especially social sciences databases and registries.
There is no mention about Grey literature search, hand search and request from experts.
The review covers a wide range of topics - an initial search of the selected databases yielded 20,000 publications (rounded), hence we do not anticipate a Grey literature and hand search. Due to the significant number of publications received in the initial search strategy and the guidelines indicating that at least two databases (Medline via Pubmed and Embase) should be included in the review, we initially did not plan to include Scopus in the search. However, given the possibility of losing important publications including a societal perspective, we decided to include this database - thank you for your consideration (additions: line 312 on the page 10;). In addition, a reference list screening is scheduled (line 314 on page 10).*

** https://guides.library.unisa.edu.au/ScopingReviews/WhereToSearch*

11. The search must include these concepts if information is expected from these areas described below. If not the search will not be able to capture the required information below.
The initial search revealed that the information in question could be extracted from the publications based on the prepared search strategy. The additional inclusion of specific search terms relating to the issues raised in the specific research questions could narrow the results of the search strategy and be counterproductive. The use of a broad search strategy, i.e. with a higher degree of generality, will yield information that answers the specific research questions.

12. What about Pregnants versus patients? What about Medical Doctors versus Nurses or allied health professionals? Please describe how this will be dealt.
The study is intended to be targeted at patients (defined as above) as the key (most important) external stakeholders in medical facilities, and medical doctors as the most important internal stakeholders [Trybou J and et al. 2014;Tryou J and et al. 2015]; other medical professions were outside the scope of this study.
We plan to extract population data while also taking into account subpopulations.(Tab. 3_ RQ5_charact. population).

- *Trybou J, Gemmel P, Van Vaerenbergh Y, Annemans L. Hospital-physician relations: the relative importance of economic, relational and professional attributes to organizational attractiveness. BMC Health Serv Res. 2014; 14:232.*
- *Trybou J, De Caluwé G, Verleye K, Gemmel P, Annemans L. The impact of professional and organizational identification on the relationship between hospital–physician exchange and customer-oriented behaviour of physicians. Human Resources for Health. 2015; 13:8.*

13. Please describe how this could help or change the results if not considered.
We have followed the Instructions for authors (BMJ Open): the required clause "…." was included in the Supplement section if patients and the public were not involved in the development of the scoping review protocol.
„*We appreciate that patient and public involvement is relatively new and may not be feasible or appropriate for all papers.* ***We therefore continue to consider papers where patients were not involved.*** *Please note that this practice is only applicable for Research Articles, Study Protocols, and Cohort Profiles."*

- *https://www.bmj.com/content/358/bmj.j3453?_gl=1*17b8ptz*_ga*MTI2NjM0NjI1Mi4xNjkzODIxNTA1*_ga_EXTSVLH45V*MTcyODU1ODU0My4xNjIuMS4xNzI4NTU4NTYwLjAuMC4xNzY4NDM2NzQ1*_fplc*NXpuQjZLbWV2RVd3eFRaN25vVXhHMGFQQzFzT3ZrJTJGMFUlMkZhUFA3SjlwSGlvenFzV0RDeEtXOTVZUTZRR2d4VTdjdDBaciUyQm5QbGFLWlFXJTJGNk8yYk4lMkJibFlwQyUyRllZOUQlMkJvelpaaGZGSU9PWnJzMFJOWjdscW8zTWJWeGdGYlElM0QlM0Q.*

This article is a non-clinical research protocol and the involvement of patients at this stage was not planned.

14. Before this section - A section on expected outcomes and the implications of this review need to be included.

This issue is addressed in section Methods and analysis in Stage 9 - Summarizing the evidence in relation to the purpose of the review, making conclusions and noting any implications of the findings (line 381-385 on the page 15) and in section Ethics and dissemination (lines 397-401 on the page 15).
This study will allow us to draw up a list of factors, whether potential or determining, that affect the use of digital health in primary healthcare, both by patients and physicians. This information is now included in the manuscript – lines: 385-392 on page 15.

Reviewer: 2
Dr. Philip Scott, University of Wales Trinity St David
Comments to the Author:
This is a well designed study with a clearly explained and justified protocol. My only reservations are
(1) that the very broad and high level nature of the "factors" may lead to very vague, woolly and diverse conclusions which may end up not adding very much,

We appreciate your evaluation and your suggestions for possible improvements and additions.

The review is indeed very broad, which inherently involves a risk of generalization; however, we hope to develop a multi-threaded matrix that will provide the key to understanding the factors that are valuable from the point of view of patients as the most important external stakeholders in a medical facility, and medical doctors as internal stakeholders.
The strategy incorporated a broad search of factors (as indicated in Table 2), using the Mesh dictionary of the Pubmed database, and Emtree of the Embase database with appropriate synonyms, so as not to miss the specified ones, such as risk factors, which the "Advanced search" is expected to capture.

(2) there is no mention of the sociotechnical perspective, which has a rich literature in this topic area (e.g. https://www.ncbi.nlm.nih.gov/pmc/articles/PMC3096986/),
The supplementary information with source links are provided in:
- page 5, lines: 134-136,
- page 6, lines: 172-177.

- *Scott PJ, Briggs JS. STAT-HI: A socio-technical assessment tool for health informatics implementations. Open Med Inform J. 2010;4:214-220. doi:10.2174/1874431101004010214*

and (3) there is no acknowledgement of some very relevant earlier work (albeit described as risk factors, rather than just 'factors affecting use'):
https://ebooks.iospress.nl/publication/51886
https://pubmed.ncbi.nlm.nih.gov/18473092/
The indicated information along with the source links are provided in:
- page 6, lines: 172-177 (Scott PJ),
- page 6, lines: 177-179 (Pare G),
- pages: 8, lines: 236-240 (Greenhalgh T; Scott P).

In addition, the scale-up was added to the list of extracted code examples in the "Process of using" section (Tab. 3)

- *Scott PJ, Briggs JS. STAT-HI: A Socio-Technical Assessment Tool for Health Informatics Implementations. The Open Medical Informatics Journal 2010;4:214–20. doi:10.2174/1874431101004010214.*
- *Paré G, Sicotte C, Jaana M, Girouard D. Prioritizing the risk factors influencing the success of clinical information system projects. A Delphi study in Canada. Methods Inf Med. 2008;47(3):251-259.*
- *Greenhalgh T, Abimbola S. The NASSS Framework - A Synthesis of Multiple Theories of Technology Implementation. Stud Health Technol Inform. 2019;263:193-204. doi:10.3233/SHTI190123*
- *Scott, P., de Keizer, N., & Georgiou, A. (Eds.) (2019). Applied Interdisciplinary Theory in Health Informatics: A Knowledge Base for Practitioners. (Studies in Health Technology and Informatics; Vol. 263). IOS Press. https://www.iospress.nl/book/applied-interdisciplinary-theory-in-health-informatics/*
